# A placental trisomy 2 detected by NIPT evolved in a fetal small Supernumerary Marker Chromosome (sSMC)

**DOI:** 10.1186/s13039-021-00535-4

**Published:** 2021-03-15

**Authors:** Justyna Domaradzka, Marta Deperas, Ewa Obersztyn, Anna Kucińska-Chahwan, Nathalie Brison, Kris Van Den Bogaert, Tomasz Roszkowski, Marta Kędzior, Magdalena Bartnik-Głaska, Alicja Łuszczek, Krystyna Jakubów-Durska, Joris Robert Vermeesch, Beata Anna Nowakowska

**Affiliations:** 1 Medical Genetics Department, The Institute of Mother and Child, Kasprzaka 17A, 01-211 Warsaw, Poland; 2 Department of Obstetrics and Gynecology, Centre of Postgraduate Medical Education, Czerniakowska 231, 00-416 Warsaw, Poland; 3 Centre for Human Genetics, KU Leuven, Herestraat 49, 3000 Leuven, Belgium

**Keywords:** Array comparative genomic hybridization, Fluorescence in situ hybridization, Karyotyping, Mosaicism, Non-invasive prenatal test, Small supernumerary marker chromosome

## Abstract

**Background:**

Non-invasive prenatal testing (NIPT) is a rapidly developing and widely used method in the prenatal screening. Recently, the widespread use of the NIPT caused a neglecting of the limitations of this technology.

**Case presentation:**

The 38-year-old woman underwent amniocentesis because of a high risk of trisomy 2 revealed by the genome-wide Non-Invasive Prenatal Test (NIPT). The invasive prenatal diagnosis revealed the mosaicism for a small supernumerary marker chromosome sSMC derived from chromosome 2. Interphase fluorescence in situ hybridization (FISH) on uncultured amniocytes revealed three signals of centromere 2 in 30% of the cells. GTG-banded metaphases revealed abnormal karyotype (47,XX,+mar[21]/46,XX[19]) and was confirmed by array comparative genomic hybridization (aCGH). Cytogenetic analyses (FISH, aCGH, karyotype) on fetal skin biopsies were performed and confirmed the genomic gain of the centromeric region of chromosome 2. In the placenta, three cell lines were detected: a normal cell line, a cell line with trisomy 2 and a third one with only the sSMC.

**Conclusion:**

Whole-genome Non-Invasive Prenatal Testing allows not only the identification of common fetal trisomies but also diagnosis of rare chromosomal abnormalities. Especially in such cases, it is extremely important to perform not only NIPT verification on a sample of material other than trophoblast, but also to apply appropriate research methods. Such conduct allows detailed analysis of the detected aberration, thus appropriate clinical validity.

## Background

Non-invasive prenatal testing (NIPT) is a rapidly developing and widely used method in the world. The NIPT resolution depends on the technique applied. Recent research shows that different NIPT approaches can be used not only to detect the most common trisomies, but also for detection of rare aneuploidies and to assess submicroscopic deletions and duplications, as well as identify monogenic diseases [1–6]. The well-documented specificity and sensitivity of NIPT for the most common trisomies (13, 18, 21) are different for each chromosome. Mackie et al. estimated specificity of 99% to 100% for 13, 21, 18 trisomies and monosomy X. The sensitivity to detect trisomy 21 reaches 99% but for trisomy 13, 18 and monosomy X it is 90%, 98% and 93% respectively [7]. Similarly study Lee et. al showed 99–100% specificity and sensitivity for trisomies 21, 13 but sensitivity for 18 is high as 92% [8]. However, in literature there is no data of NIPT sensitivity and specificity for other chromosomes.

Moreover discordant results between the NIPT and the fetal karyotype have been reported. This can be due to the fact that the fetal cell-free DNA (cfDNA) in the maternal blood is derived from the cytotrophoblastic cells of the placenta [9] and confined placental mosaicism (CPM) can yield a discrepancy between NIPT and amniotic fluid analysis. CPM are observed in about 1–2% of chorionic villus samplings (CVS) [10]. Additionally, twin pregnancies, vanisching twing, maternal copy number variations (CNV), maternal mosaicism, as well as maternal malignancies but also low-level imbalance-mosaicism examined chromosomes are the most commonly reported reasons for discordance [11–19].

Therefore, in the case of fetal abnormalities identified during the ultrasound screening, invasive test instead of NIPT is recommended [20]. Here we report a discrepancy between the result of NIPT and the final genetic diagnosis. The indication for the prenatal examination in this case was a high risk of trisomy 2 revealed by NIPT test while in invasive diagnostic mosaicism of a small supernumerary marker chromosome (sSMC) derived from chromosome 2 has been detected.

### Case presentation and methods

A 38-year old woman came to the clinic because of increased risk of trisomy 21 (T21 = 1/291) calculated based on the combined test. The ultrasound test did not reveal any abnormalities. The patient conducted NIPT at the 14th week of gestation, which revealed a high risk of trisomy 2. Amniocentesis was performed at 17th week of gestation. The amniotic fluid was aliquoted in order to perform FISH, aCGH and conventional karyotyping analysis. A fetal skin and placental were taken to perform FISH and aCGH on uncultured cells, and conventional karyotyping on cultured cells.

## Methods

### Fluorescence in situ hybridization (FISH)

Interphase FISH analysis was performed on uncultured amniotic fluid cells using commercially available DNA probe for region 2p11.1-q11.1 (Spectrum Red centromere of chromosome 2 probe, locus D2Z2) (Chromosome 2 Alpha Satellite Probe—Cytocell) and probe for region Xp11.1-q11.1 (Spectrum Green centromere of chromosome X probe, locus DXZ1) (Chromosome X Alpha Satellite Probe—Cytocell) as a control. Fetal skin and placenta cells were examined with the same probe for chromosome 2 (Chromosome 2 Alpha Satellite Probe—Cytocell) and locus specific probe RP11-676E9 from bacterial artificial chromosome (BAC) clone located at cytoband 2p21 (chr2:45,579,001-45,773,026; GRCh37). The position of the BAC clone was taken from the UCSC Genome Browser. DNA was labelled with Spectrum Green-dUTP (Vysis), using the Vysis nick-translation kit (Vysis). Labelling of probe was done as described previously by Merscher et al. [21].

### Array comparative genomic hybridization (aCGH)

DNA was extracted directly from amniotic fluid cells and from fetal skin cells. Microarray was performed using CytoSure Constitutional v3 (8 × 60 k), (Oxford Gene Technology, GRCh37/hg19) according to procedures described by Bartnik et al. [22]. Scanned images were quantified using Agilent Feature Extraction software (v10.0). The CytoSure (Oxford Gene Technology) software was used for chromosomal microarray analysis. All genomic coordinates are based on the March 2006 assembly of the reference genome (NCBI37/hg19).

### Karyotype analysis

Analysis of GTG-banded metaphases at approximately 400-band resolution in amniocytes and skin fibroblasts after in situ culturing was performed according to the standard protocol.

## Result

**Fig. 1:**
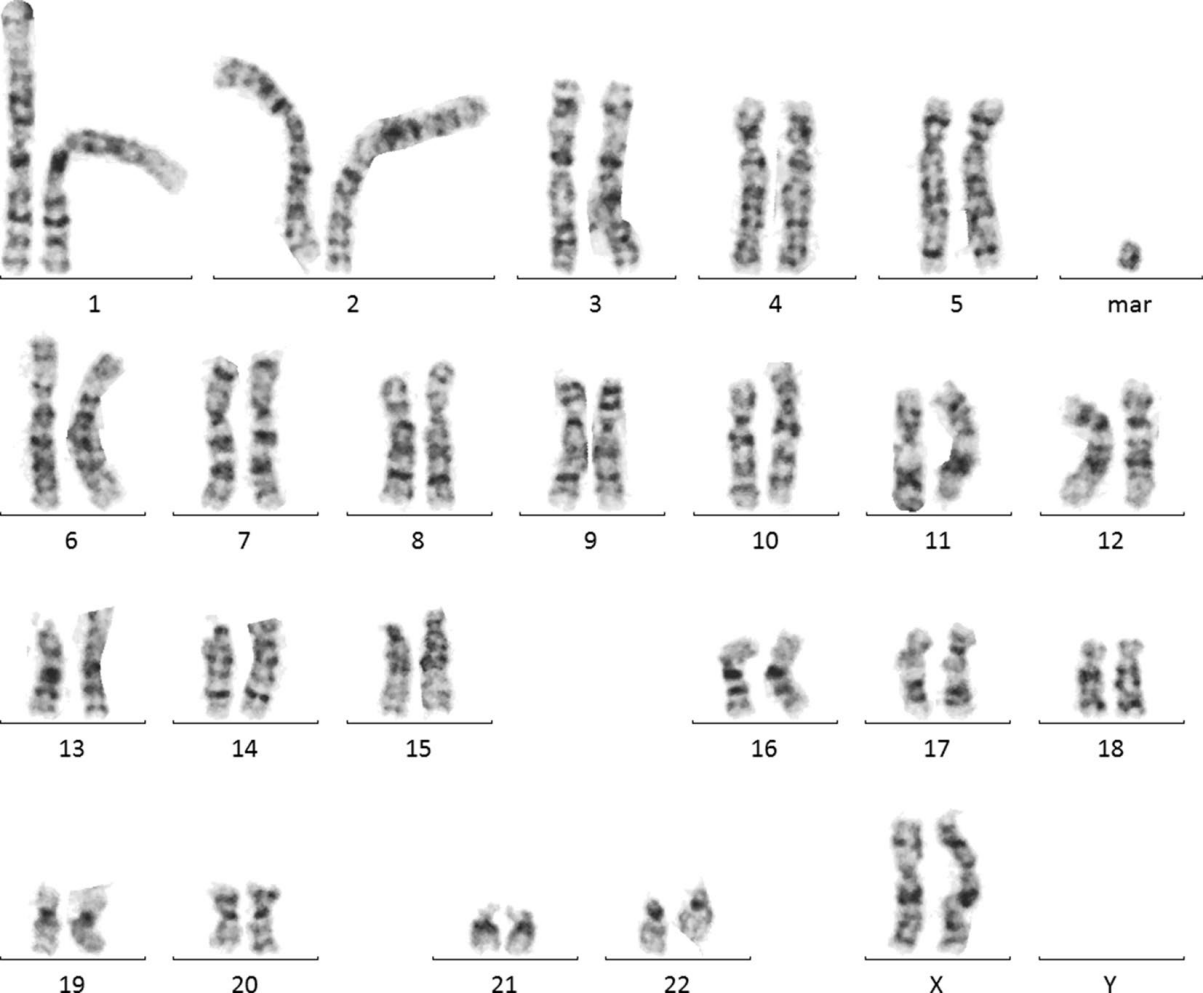
A GTG-banded metaphase (47,XX, + mar) at 400-band resolution carrying sSMC

**Fig. 2:**
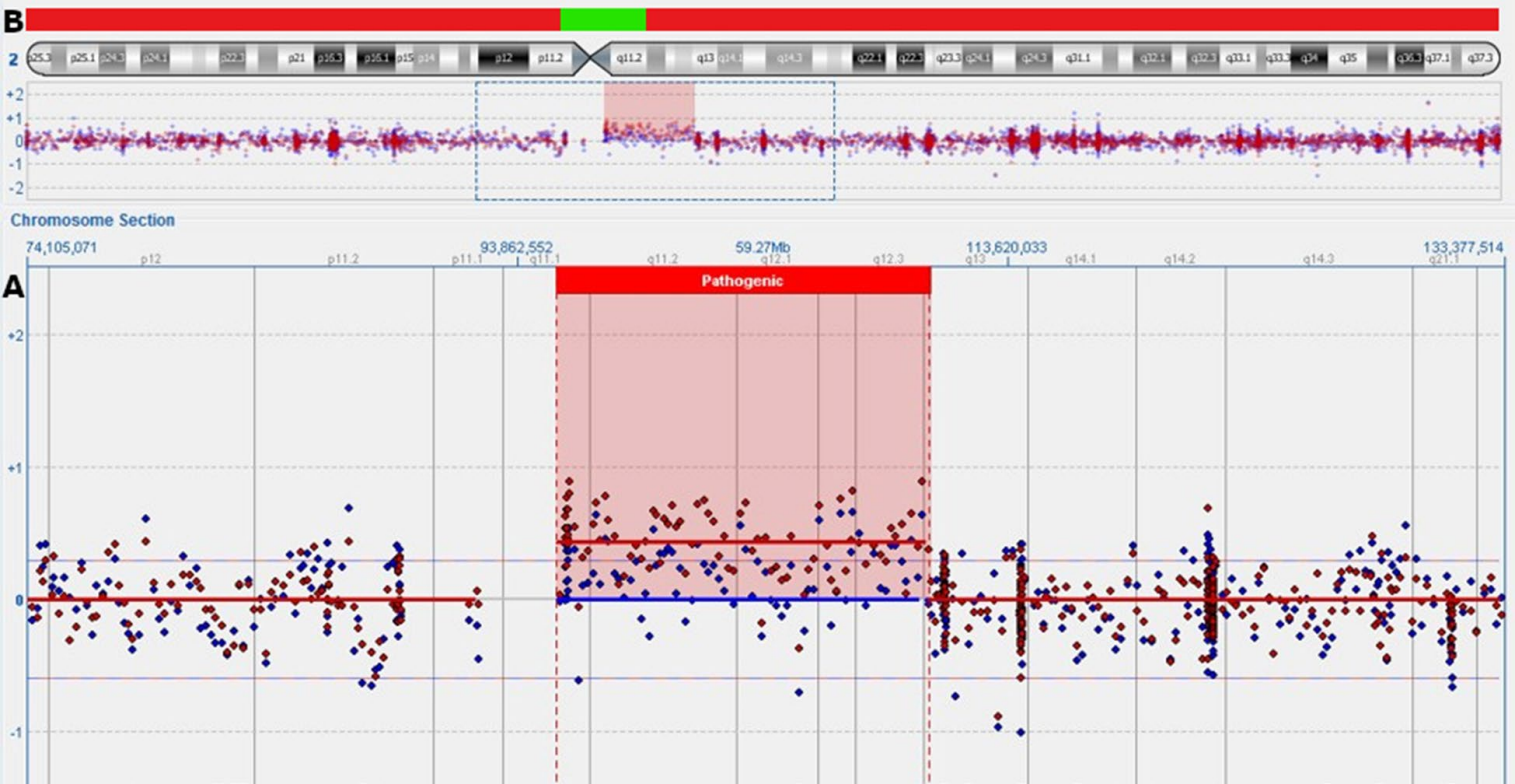
**a** Array CGH analysis of amniocytes (blue plot) and fetal skin cells (red plot) showing 14,83 Mb genomic gain of chromosome 2q11.1q13 (95420515_1102553160; area in red shadow). **b** The green line marks schematic cytogenetic depiction of probably non-dosage sensitive pericentric region of chromosome 2. The red line marks schematic cytogenetic depiction of chromosome 2 region with clinical relevance [23]

**Fig. 3:**
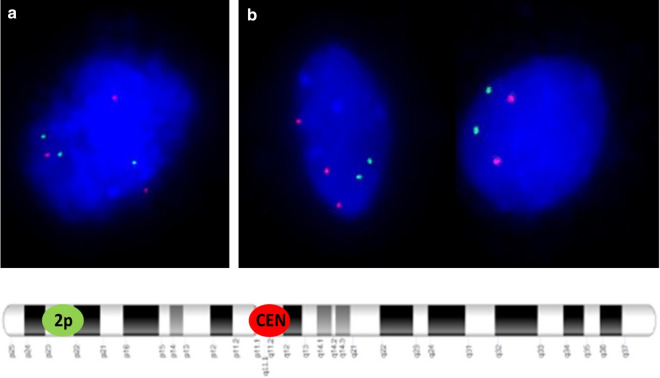
Interphase FISH analysis of uncultured placenta (**a**) and fetal skin (**b**) cells showing red signals from centromeres of chromosome 2 and green signals from specific locus in p arm of chromosome 2. Loci for FISH probes on chromosome 2 are presented in the scheme below

Interphase FISH analysis performed on amniotic fluid showed three signals for the centromere of chromosome 2 in 45 cells and two signals in 105 cells, indicating 30% mosaicism for trisomy 2 in female fetus. However, based on the interphase FISH, structural chromosomal abnormalities cannot be identified. Analysis of GTG-banded metaphases revealed a 47,XX,+mar[21]/46,XX[19] karyotype indicating the presence of a marker chromosome in 52% of the analyzed metaphases. In order to determine the genetic content of the sSMC, aCGH was performed. Array CGH indicated a pericentromeric gain of 14.83 Mb in chromosomal region 2q11.1q13 (95420515_1102553160). Based on the morphology of the marker detected in GTG-banding (Fig. 1) and molecular results, we concluded that the marker was a small supernumerary ring chromosome (sSRC) derived from chromosome 2. Based on Liehr online database [23] (the panel B in Fig. 2), the region is known as pathogenic, causing dysmorphism, developmental delay, brain malformations, heart defect, hypotonia, mental retardation, finger or toe/foot malformations, growth retardation, kidney problems/ malformations and omphalocele or situs inversus. Following detailed genetic counselling, the parents decided to terminate the pregnancy. Ultrasound examination and postnatal autopsy at the 22nd week of gestation revealed a female fetus with growth restriction but without any further malformations. Fetal skin an placental tissue were sampled to perform further cytogenetic examinations. Array CGH of skin cells (red plot in Fig. 2) showed the same 14.83 Mb gain of chromosome 2q11.1q13 (95420515_1102553160). However, this time the plot showed higher level of mean log ratio (0.44 on average), compared to results from the amniotic fluid cells (0.19 on average), indicating higher level of mosaicism in skin cells. FISH analysis of fetal skin cells (Fig. 3b) showed three signals of centromere 2 in 84 of 132 analyzed nuclei, indicating 64% of mosaicism for the sSMC. The placenta (Fig. 3a) showed the presence of three cell lines: 39% of scored nuclei had a normal number of signals from chromosome 2, 40% presented trisomy of the whole chromosome 2 and 21% carried the sSMC. Finally, cytogenetic analysis by conventional karyotype of cultured skin and placental cells revealed 30% and 33% mosaicism of the sSMC respectively (Table 1), however, no trisomy of whole chromosome 2 was detected.

**Table 1:** Summarized results from FISH and GTG-banding scoring

Biological material	Treatment	Method of analyses	Results	Mosaicism [%]
Amniotic fluid	Uncultured	FISH	nuc ish(D2Z2×3)[45/150]	30
	Cultured	GTG	mos 47,XX,+mar[21]/46,XX[19]	52
Fetal skin	Uncultured	FISH	nuc ish(D2Z2×3)[84/132]	64
	Cultured	GTG	47,XX,+mar[15]/46,XX[35]	30
Placenta	Uncultured	FISH	nuc ish(D2Z2,RP11-676E9)×3[45/112]/ (D2Z2×3,RP11-676E9×2)[23/112]	40/21
	Cultured	GTG	47,XX,+mar[6]/46,XX[12]	33

*GTG* Giemsa trypsin G-banding, *FISH* fluorescence in situ hybridization, *Nuc ish* nuclear in situ hybridization, *Mos* mosaic, *Mar* marker chromosome

## Discussion and conclusions

Herein, we report the prenatal diagnosis and molecular cytogenetic characterization of the mosaicism for sSMC derived from chromosome 2. The indication for the prenatal examination in this case was a high risk of trisomy 2 revealed by NIPT test. Our case underlines the screening nature of the NIPT test and also demonstrates that performing only the FISH analyses in uncultured amniocytes, as the most rapid way to verify the NIPT result, would be insufficient for the correct diagnosis. Therefore, bearing in mind that the fetal and placental karyotypes might be different, a good follow-up of abnormal NIPT results is necessary, but as the investigation showed, techniques as well as the tested tissue should be properly selected.

Chromosomal mosaicism can be associated with a wide spectrum of phenotypes extending from apparently normal to severe or lethal. Hsu and coauthors [24] summarized the outcome of 11 fetuses with whole chromosome trisomy 2 detected in mosaicism, which ranged from 4 to 33% and has been confirmed in other tissues. Within these cases, 1 with the lowest percentage of abnormal cells (4% in the amniocytes but without trisomy in blood and placenta) resulted in an apparently normal livebirth; 1 newborn presented IUGR, 1 had IUGR and multiple anomalies, 3 stillbirths or intrauterine deaths, and 4 in elective terminations (11–33% mosaic cells, all with abnormal findings).

Clinical consequences in cases of mosaicism of partial trisomy can be harder to predict. Marker chromosomes derivative from chromosome 2, described in the literature, have been associated with multiple clinical consequences. Children diagnosed after birth might present: developmental delay, brain malformations, seizures, heart defect, kidney malformations, hypotonia, mental retardation, growth retardation, microcephaly [23]. However, little is known about prenatal ultrasound abnormalities in such cases.

Microarray analysis defined the gain of 14.83 Mb in the 2q11.1q13 region. Our case is highly similar to a case reported by Riegel and Schinzel [25]. Authors described a 4-year-old boy with multiple clinical features and a duplicated segment of 2q11.1-q13.2 presented in all analyzed cells. Low birth weight (< 10th centile), a left cleft lip with cleft palate and natal left upper incisor were noted at birth. Consecutive examination revealed mental retardation, low set ears, irregular teeth, cryptorchidism and epilepsy. However, also in this case, three ultrasound routine examinations performed prenatally did not disclose abnormal findings. The difference in the clinical presentation between our case and the patient described by Riegel and Schinzel [25], might be due to the percentage of the level of normal cells, but also could be because of different impact of the simple region duplication in the genome versus marker chromosome.

Furthermore, cytogenetic discrepancy between results of uncultured fetal cells (skin 64%, amniocytes 30%) and cultured cells (skin 30%, amniocytes 52%) provides an additional challenge in genetic counselling. FISH analyses in uncultured skin cells presented higher percentage of mosaicism (64%) than was found later in cultured skin cells (30%). The discrepancy has also been observed between uncultured and cultured placenta cells. In uncultured placenta we observed three cell lines; with a normal number of signals from chromosome 2 (39%), trisomy of whole chromosome 2 (40%), and 21% of cells carrying the sSMC. Whereas cultured placenta did not reveal the whole trisomy 2. The variations in cell numbers carrying an abnormality before and after cultivation are compatible with results of other authors and presumably stem from a selection against the trisomic cells after long-term culture [26]. Different values of mosaicism indicated in uncultured (30%) and cultured (52%) amniocytes can be caused by the contamination of amniotic fluid with maternal cells. Moreover, the number of available for analysis metaphases was relatively low (n = 40) in comparison to uncultured nuclei analyzed with FISH (n = 150). The fact that the abnormal cells may divide slower and undergo apoptosis more easily causes an increase in the proportion of normal cells, should be taken into consideration during genetic consultation [27].

In the presented case, based on FISH and GTG-banding results, the sSMC was in fact a small ring chromosome. There is a high probability that this ring chromosome formation is caused by low repetitive elements present in the pericentric region of chromosome 2 [28, 29].

We have observed a discrepancy between the indication for invasive testing, and the final genetic diagnosis results. However, the presence of the trisomy of whole chromosome 2 was confirmed in the placenta. It is probable that the marker chromosome has arisen from trisomic embryo cells, which existed at an initial stage of development. The presence of the marker chromosome mosaicism in subsequent studies points to the existence of a functional trisomy rescue mechanism in this case.

A genetic counselling of fetuses with mosaicism is especially problematic because of the relatively poor phenotypic data and time-limitation. Considering the fate of the pregnancy in the case of detecting abnormalities, NIPT can be assessed as a screening test, and should be accompanied by an ultrasound examination. Hence, invasive diagnosis is necessary to confirm the non-invasive results. In the presented case, it should be emphasized that there is a discrepancy between the result of the NIPT study and the results of the genetic diagnostic tests performed in the fetal tissues. Therefore it is crucial to choose the most suitable investigation strategy in order to perform the most rapid genetic diagnosis.

## Data Availability

All data generated or analysed during this study are included in this published article.

## Abbreviations

NIPT: non-invasive prenatal testing
FISH: fluorescence in situ hybridization
aCGH: array comparative genomic hybridization
sSMC: small supernumerary marker chromosome
cfDNA: cell-free DNA
IUGR: intrauterine growth restriction
sSRC: small supernumerary ring chromosome
GTG: Giemsa trypsin G-banding
Nuc ish: nuclear in situ hybridization
Mos: mosaic
Mar: marker chromosome
Mb: mega base pairs
CVS: chorionic villus sampling
CPM: confined placental mosaicism
CNV: copy number variations
